# The Effect of Statins on Blood Gene Expression in COPD

**DOI:** 10.1371/journal.pone.0140022

**Published:** 2015-10-13

**Authors:** Ma’en Obeidat, Nick Fishbane, Yunlong Nie, Virginia Chen, Zsuzsanna Hollander, Scott J. Tebbutt, Yohan Bossé, Raymond T. Ng, Bruce E. Miller, Bruce McManus, Stephen Rennard, Peter D. Paré, Don D. Sin

**Affiliations:** 1 The University of British Columbia Centre for Heart Lung Innovation, St Paul’s Hospital, Vancouver, BC, Canada; 2 Prevention of Organ Failure (PROOF) Centre of Excellence, Vancouver, BC, Canada; 3 Respiratory Division, Department of Medicine, University of British Columbia, Vancouver, BC, Canada; 4 Institut universitaire de cardiologie et de pneumologie de Québec, Department of Molecular Medicine, Laval University, Québec, Canada; 5 Respiratory Therapy Area Unit, GlaxoSmithKline R&D, King of Prussia, Pennsylvania, United States of America; 6 Division of Pulmonary and Critical Care Medicine, University of Nebraska Medical Center, Omaha, Nebraska, United States of America; University of Manitoba, CANADA

## Abstract

**Background:**

COPD is currently the fourth leading cause of death worldwide. Statins are lipid lowering agents with documented cardiovascular benefits. Observational studies have shown that statins may have a beneficial role in COPD. The impact of statins on blood gene expression from COPD patients is largely unknown.

**Objective:**

Identify blood gene signature associated with statin use in COPD patients, and the pathways underpinning this signature that could explain any potential benefits in COPD.

**Methods:**

Whole blood gene expression was measured on 168 statin users and 451 non-users from the ECLIPSE study using the Affymetrix Human Gene 1.1 ST microarray chips. Factor Analysis for Robust Microarray Summarization (FARMS) was used to process the expression data. Differential gene expression analysis was undertaken using the Linear Models for Microarray data (Limma) package adjusting for propensity score and surrogate variables. Similarity of the expression signal with published gene expression profiles was performed in ProfileChaser.

**Results:**

25 genes were differentially expressed between statin users and non-users at an FDR of 10%, including *LDLR*, *CXCR2*, *SC4MOL*, *FAM108A1*, *IFI35*, *FRYL*, *ABCG1*, *MYLIP*, and *DHCR24*. The 25 genes were significantly enriched in cholesterol homeostasis and metabolism pathways. The resulting gene signature showed correlation with Huntington’s disease, Parkinson’s disease and acute myeloid leukemia gene signatures.

**Conclusion:**

The blood gene signature of statins’ use in COPD patients was enriched in cholesterol homeostasis pathways. Further studies are needed to delineate the role of these pathways in lung biology.

## Introduction

Chronic obstructive pulmonary disease (COPD) is characterized by chronic irreversible airflow limitation that is often accompanied by systemic inflammation and comorbidities [1,2]. COPD currently ranks as the fourth leading cause of death, and is predicted to become the third by 2020 [3]. Statins are widely used lipid-lowering agents that act through inhibiting 3-hydroxy-3-methylglutaryl coenzyme A (HMG-CoA) reductase, the rate-limiting step of hepatocyte cholesterol synthesis. Statins reduce the risk of cardiovascular morbidities and mortality [4]. Statins are also reported to have pleiotropic effects including anti-inflammatory, anti-oxidant, anti-thrombogenic and neuroprotective properties [5,6].

A number of retrospective studies have shown that statins play a beneficial role in COPD. The endpoints in these studies included reductions in the rate of hospitalization [7], lung function decline[8], and death [9]. Meta-analysis approaches have further supported the beneficial role of statins on outcomes among COPD patients [10–12]. A number of epidemiological studies reported significant correlations between serum lipids and lung function measures. In the Third National Health and Nutrition Examination Survey (NHANES), forced expiratory volume in one second (FEV_1_) was positively related to LDL and negatively related to HDL [13]. Additionally, oxidized LDL levels were increased in COPD patients and were associated with lung function, inflammation, and oxidative stress [14]. Conversely, associations in the opposite direction were reported in the Multi- Ethnic Study of Atherosclerosis (MESA) study, in which HDL levels were negatively associated with FEV_1_/FVC ratio and percent emphysema [15]. Increased HDL levels have also been reported in patients with severe COPD [16,17].

At the molecular level, many genes involved in cholesterol homeostasis and transport are expressed in the lungs [18,19], and *in vitro* and *in vivo* studies have demonstrated the importance of cholesterol homeostasis to lung physiology (reviewed in [20,21]). Additionally, in animal models that mimic smoke-induced lung injury, statins were shown to inhibit airway epithelial injury [22] and to reverse smoke-induced pulmonary hypertension and prevent emphysema [23].

Recently, the largest clinical trial to date showed that simvastain 40mg/day did not affect exacerbation rates or the time to a first exacerbation in patients with COPD who were at high risk for exacerbations[24]. However, because this study excluded all patients with indications for statins such as those with cardiovascular comorbidities, diabetes, and even preclinical diabetes (i.e. elevated hemoglobin A1C), the results may lack generalizability to a “real-world” setting in which 20 to 40% of COPD patients are taking statins for cardiovascular indications. The impact of statins, beyond those on the cardiovascular system, in this setting is largely unknown.

The objective of the current study was to investigate the effects of statins on blood gene expression in a cohort of well-phenotyped COPD patients and to determine whether the changes in blood gene expression could suggest potential benefits in COPD.

## Methods

### Study participants

Study subjects were a subset of those in the ECLIPSE (Evaluation of COPD Longitudinally to Identify Predictive Surrogate Endpoints) cohort[25]. ECLIPSE is a non-interventional, multicentre, longitudinal prospective three-year study of individuals with COPD and control subjects. Blood was collected in PAXGene tubes and frozen at -80°C. A total of 619 subjects were selected for studying prognostic signatures of frequent COPD exacerbations. Out of the 619 subjects, 168 were statin users. The ECLIPSE study was conducted in accordance with the Declaration of Helsinki and good clinical practice guidelines, and has been approved by the relevant ethics and review boards at the participating centres. At the University of British Columbia (UBC), the Research Ethics Board (REB) number is H11-00786. Study participants received written informed consent. Patient information was anonymized and de-identified prior to the analysis. ECLIPSE study was funded by GlaxoSmithKline, under ClinicalTrials.gov identifier NCT00292552 and GSK No. SCO104960.

### Microarray data processing

Total RNA was extracted on QIAcube (Qiagen Inc) from the 64 PAXgene blood using the PAXgene Blood miRNA kit from PreAnalytix (Cat. #763134) according to manufacturer’s instructions. RNA was amplified and hybridized overnight to the Affymetrix Human Gene 1.1 ST array plates at the TSRI DNA Array Core Facility, Scripps Research Institute (La Jolla, CA). Array plates were scanned using the Affymetrix GeneTitan MC Scanner (Affymetrix Inc.) with default settings. Quality control of the microarray data was performed using oligo (oligo Bioconductor package)[26] and RMA Express (http://rmaexpress.bmbolstad.com/)[27]. Factor Analysis for Robust Microarray Summarization (FARMS Bioconductor package) [28] was used to background correct, normalize, and summarize the data and filter out non-informative probe sets. The raw expression data is available on the NCBI Gene Expression Omnibus (GEO) under accession number GSE71220, http://www.ncbi.nlm.nih.gov/geo/query/acc.cgi?acc=GSE71220.

### Differential gene expression analysis

Differential gene expression analysis was performed using the Linear Models for Microarray Data (Limma) Bioconductor package[29]. To adjust the differential expression analysis for potential confounders, we created a propensity score and used the score as a covariate in Limma. The propensity score assigns a probability for being a statin user based on observed baseline characteristics [30]. Thus, for each patient, the propensity score is the probability that that patient would have been assigned to the statin group rather than control, given their measured characteristics. Using the StepAIC() function from the R stats package, ten covariates were identified from a collection of available phenotypes using a bidirectional stepwise regression with Akaike information criterion (AIC); these were age, asthma, smoking, BMI, FEV_1_, FEV_1_% predicted, inhaled corticosteroid use, hypertension, diabetes and cardiovascular history. Next, propensity score was computed for each individual using a logistic regression model with those ten covariates using the glm() function from the R stats package. Finally, the resulting propensity score was used as a covariate when modeling the statin effect on gene expressions using the R package limma.

Testing for differential gene expression using the propensity score produced a distribution of inflated test statistics. To correct for this, Surrogate Variable Analysis (SVA) [31] was used to identify components of expression heterogeneity and unknown sources of variation. SVA analysis was performed in the sva Bioconductor package [32]. The differential expression analysis in limma was then adjusted for propensity score, neutrophil percentage, monocyte percentage and for the 39 significant surrogate variables found in SVA.

### Pathway enrichment analysis

Genes differentially expressed with respect to statin use were tested for enrichment in biological processes and pathways using the web-based gene set anaLysis toolkit (WebGestalt) [33]. Gene symbols were used as input for WebGestalt and tested for enrichment in Kyoto Encyclopedia of Genes and Genomes (KEGG) pathways [34] and Gene Ontology (GO) processes [35]. Enrichment was undertaken using a hypergeometric model [36] and adjusting for multiple testing using the Benjamini Hochberg (BH) method [37].

### ENCODE transcription factor enrichment analysis

To gain insight into what transcription factors are enriched in the promoters and 5`regions of genes within modules differentially expressed with statin use, we used the encyclopedia of the DNA elements (ENCODE) Chromatin Immunoprecipitation Sequencing (ChIP-Seq) Significance Tool [38]. Gene symbols were used as input, and “all IDs” was selected as the background region for the hypergeometric test. A window of 500bp to 5000bp length from the transcription start sites was used for enrichment as provided by the software, and used all cell lines as input.

### Similarity with other gene expression profiles

To gain insights into the biological significance of the statins-associated gene expression profile, the ProfileChaser online tool [39] was used to identify gene expression datasets from the Gene Expression Omnibus with similar expression profiles. ProfileChaser scores the similarity between two differentially expressed profiles (one supplied by the user and the comparison profile) using a weighted Pearson’s correlation. A null distribution for this measure is computed from the correlation coefficients of all experimental comparisons available in the ProfileChaser database, allowing for the calculation of a conservative false discovery rate (FDR) for each retrieved result. The expression values of statins-associated genes at nominal P values (*P*< 0.05) were used as input for ProfileChaser.

ProfileChaser output was manually filtered to keep only experiments that have more than 20 samples, and experiments performed in whole blood or blood-derived cell types.

### Statistical analysis software

All statistical analyses were performed using R version 3.0.1 (http://www.r-project.org/). The following Bioconductor packages were used for microarray data processing: oligo[26], RMA Express [27], and FARMS [28]. The SVA package [32] was used for the surrogate variable analysis, and the differential expression was performed using the Limma package [29].

## Results

In this study, 168 statin users and 452 non- users were included. The demographics of study participants are presented in Table 1. The two groups were different with respect to their age, gender, pack-years of smoking and as expected, diabetes and cardiovascular history. Applying FARMS filtering; the resulting blood gene expression dataset included 11,523 probesets that map to 7185 unique genes.

**Table 1:** Study participants’ demographics.

	Statin (n = 168)	Non-statin (n = 451)	P value
**Age**	65.2 (0.42)	63.0 (0.31)	3.48E-05
**Gender Male: Female**	124: 44	284: 167	0.011
**Smoking status:**			
Current smoker	40 (24%)	91 (20%)	0.4
Former smoker	123 (73%)	338 (75%)	
Never smoked	5 (3%)	22 (5%)	
**Pack/years of smoking**	51.5 ± 2.23	44.2 ± 1.23	4.39E-03
**FEV**_**1**_ **%predicted**	53.9 (1.73)	55.7 (1.14)	0.38
**Controls (n = 57)**	13	45	
Former smoker	8	23	0.73
Never smoker	5	22	
**GOLD stage**			
2	70	194	0.78
3	65	167	
4	20	45	
**Cardiovascular history Yes: No**	129: 39	205: 246	3.54E-12
**Diabetes Yes: No**	37: 131	31: 420	8.32E-08
**Inhaled corticosteroids Yes: No**	110:57	308:108	0.048
**Long acting beta agonists Yes: No**	108: 59	289: 127	0.26
**Long acting anticholinergic Yes: No**	95:72	217:199	0.30

Continuous variables are shown as mean+ standard error of man (SEM). Difference in demographics between statin users and non-users was calculated using Student’s t-test for continuous variables and Chi-square test for categorical variables

**Fig 1:**
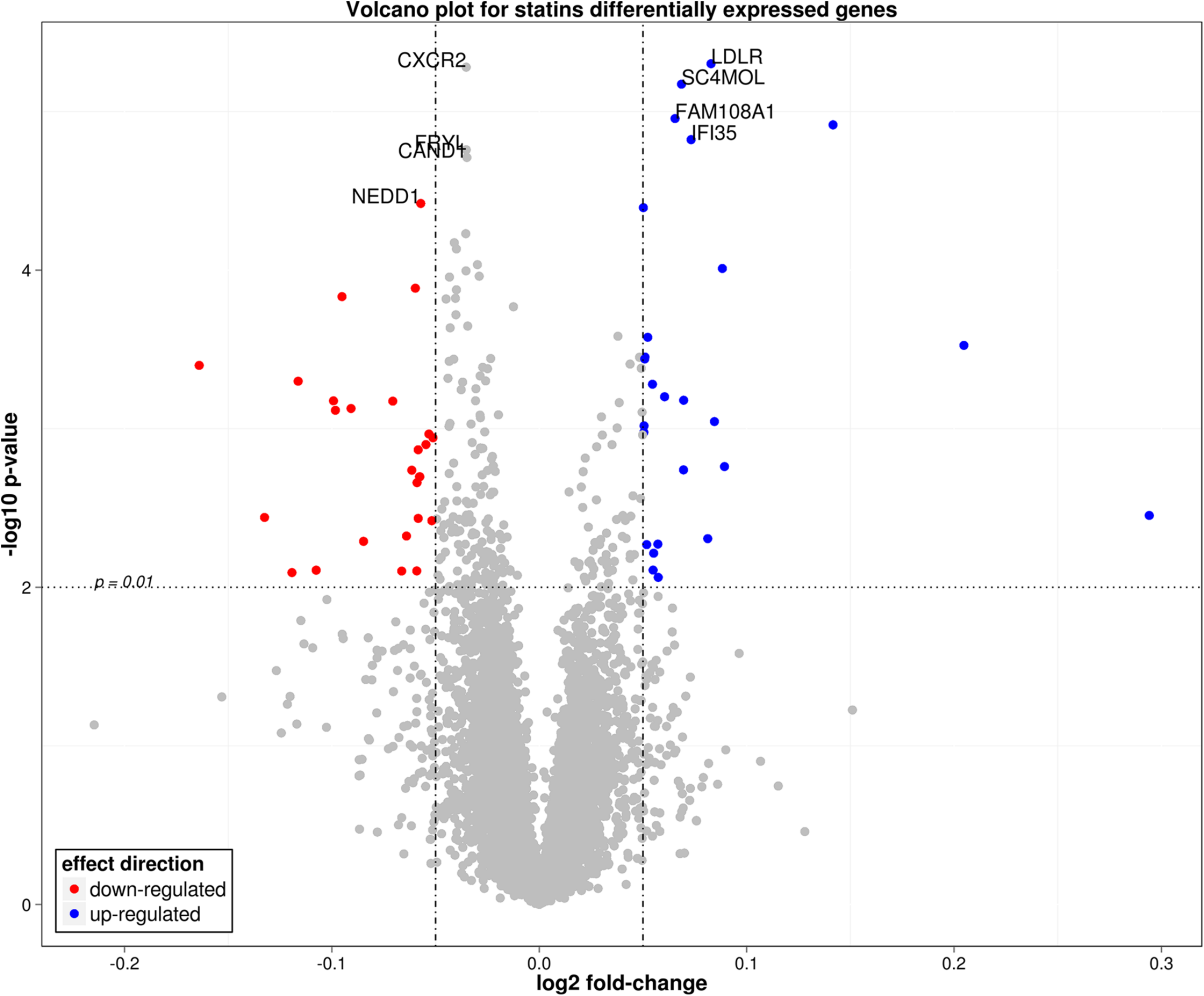
Volcano plot of gene differentially expressed between statin users and non-users. The plot shows the log fold-change on the X-axis versus the unadjusted P values (on the–log10 scale) on the Y-axis. The blue and red dots represent genes that showed fold change in either direction that is greater than 0.05 and have unadjusted P value <0.01. Genes that had an FDR-adjusted P value less than 0.05 for differential expression are annotated on the graph.

The results of differential gene expression analysis with respect to statin use adjusted for propensity score and surrogate variables are shown in Table 2. The table shows 25 and 8 genes were differentially expressed at Benjamini Hochberg FDR less than 10 and 5%, respectively. The most significant differentially expressed gene was the low-density lipoprotein receptor (*LDLR*) gene, which was up-regulated among COPD patients using statins (log2 fold-change of 0.097). The second gene was the the chemokine (C-X-C- motif) receptor 2 (*CXCR2*, log2 fold-change of -0.04), which was down-regulated among statin users. This was followed by sterol-C4-methyl oxidase-like (*SC4MOL*, log2 fold-change of 0.07) and family with sequence similarity 108, member A1 (*FAM108A*, log2 fold-change of 0.07), both up-regulated among statin users. Fig 1 shows a volcano plot of the results, annotating genes differentially expressed at FDR <5%.

**Table 2:** Statins’ differentially expressed genes at 10% FDR.

Gene Symbol	Gene Name	Log FC	P value	adj. P value
LDLR	low density lipoprotein receptor	0.083	4.99E-06	0.026
CXCR2	chemokine (C-X-C motif) receptor 2	-0.035	5.24E-06	0.026
SC4MOL	sterol-C4-methyl oxidase-like	0.069	6.72E-06	0.026
FAM108A1	family with sequence similarity 108, member A1	0.065	1.11E-05	0.028
IFI35	interferon-induced protein 35	0.073	1.50E-05	0.028
FRYL	FRY-like	-0.035	1.74E-05	0.028
CAND1	cullin-associated and neddylation-dissociated 1	-0.035	1.95E-05	0.028
NEDD1	neural precursor cell expressed, developmentally down-regulated 1	-0.057	3.80E-05	0.042
CCDC76	coiled-coil domain containing 76	-0.035	5.88E-05	0.057
TROVE2	TROVE domain family, member 2	-0.041	6.71E-05	0.059
FRY	furry homolog (Drosophila)	-0.040	7.35E-05	0.060
TIAL1	TIA1 cytotoxic granule-associated RNA binding protein-like 1	-0.030	9.23E-05	0.067
DHCR24	24-dehydrocholesterol reductase	0.088	9.76E-05	0.067
SACM1L	SAC1 suppressor of actin mutations 1-like (yeast)	-0.035	0.000101	0.067
STAG3L4	stromal antigen 3-like 4	-0.029	0.000109	0.067
TOPORS	topoisomerase I binding, arginine/serine-rich, E3 ubiquitin protein ligase	-0.043	0.000111	0.067
MYLIP	myosin regulatory light chain interacting protein	-0.060	0.00013	0.073
INTS7	integrator complex subunit 7	-0.040	0.000133	0.073
ABCG1	ATP-binding cassette, sub-family G (WHITE), member 1	-0.095	0.000147	0.073
FOSL2	FOS-like antigen 2	-0.040	0.00015	0.073
TYW3	tRNA-yW synthesizing protein 3 homolog (S. cerevisiae)	-0.045	0.000152	0.073
SGPP1	sphingosine-1-phosphate phosphatase 1	-0.012	0.00017	0.078
SNORD4B	small nucleolar RNA, C/D box 4B	-0.040	0.000191	0.085
GLS	glutaminase	-0.035	0.000225	0.095
CROT	carnitine O-octanoyltransferase	-0.043	0.000231	0.095

LogFC: log 2 fold change. Adj.p.value: Benjamini Hochberg Adjusted P value

At the pathway enrichment level, the 25 genes with FDR <10% were significantly enriched in a number of GO processes summarized in Table 3. Most of these processes were related to cholesterol homeostasis and metabolism. The most significant GO processes included cholesterol homeostasis, sterol homeostasis, regulation of plasma lipoprotein particle levels, lipid homeostasis, and sterol metabolic process. There were no significant KEGG pathways enriched among the 25 genes.

**Table 3:** GO biological processes’ enrichment among statin differentially expressed genes.

Biological Process	P values	Adjusted P value	Genes in this pathway
regulation of plasma lipoprotein particle levels	3.63E-05	0.004	*MYLIP* / *LDLR* / *ABCG1*
cholesterol homeostasis	7.48E-05	0.004	*MYLIP* / *LDLR* / *ABCG1*
sterol homeostasis	7.48E-05	0.004	*MYLIP* / *LDLR* / *ABCG1*
lipid homeostasis	2.00E-04	0.007	*MYLIP* / *LDLR* / *ABCG1*
sterol metabolic process	6.00E-04	0.012	*LDLR* / *DHCR24* / *ABCG1*
protein catabolic process	6.00E-04	0.012	*TOPORS* / *MYLIP* / *LDLR* / *DHCR24* / *ABCG1*
cholesterol metabolic process	5.00E-04	0.012	*LDLR* / *DHCR24* / *ABCG1*
lipid biosynthetic process	7.00E-04	0.013	*SACM1L* / *LDLR* / *DHCR24* / *ABCG1* / *SGPP1*
alcohol metabolic process	8.00E-04	0.013	*LDLR* / *DHCR24* / *ABCG1* / *SGPP1*
lipid modification	0.001	0.014	*SACM1L* / *CROT* / *ABCG1*
cellular metabolic process	0.0027	0.035	*CROT* / *LDLR* / *CXCR2* / *DHCR24* / *TOPORS* / *FOSL2* / *SGPP1* / *CAND1* / *GLS* / *TIAL1* / *TYW3* / *FRY* / *INTS7* / *FRYL* / *SACM1L* / *MYLIP* / *TROVE2* / *ABCG1*
lipid metabolic process	0.0033	0.040	*SACM1L* / *CROT* / *LDLR* / *DHCR24* / *ABCG1* / *SGPP1*
cellular lipid metabolic process	0.004	0.044	*SACM1L* / *CROT* / *LDLR* / *ABCG1* / *SGPP1*
single-organism catabolic process	0.005	0.048	*GLS* / *CROT* / *LDLR*
small molecule catabolic process	0.0049	0.048	*GLS* / *CROT* / *LDLR*
organic substance metabolic process	0.0055	0.050	*CROT* / *LDLR* / *CXCR2* / *DHCR24* / *TOPORS* / *FOSL2* / *SGPP1* / *CAND1* / *GLS* / *TIAL1* / *TYW3* / *FRY* / *INTS7* / *FRYL* / *SACM1L* / *MYLIP* / *TROVE2* / *ABCG1*

Adjusted P value: The Benjamini Hochberg Adjusted P value. No. of genes: number of genes in this pathway present among the 18 genes tested.

The 25 genes that met the 10% FDR for differential expression were tested for enrichment in certain transcription factors binding sites as determined from ChIP-Seq experiments from the ENCODE project. The 18 genes were enriched for the transcription factor sterol regulatory element binding protein 2 (SREBP-2), at FDR = 1.12x10^-4^.

Finally, the expression profiles of 707 differentially expressed genes identified in this study at nominal P values (*P*<0.05) were compared with publically available experiments in Gene Expression Omnibus (GEO) to gain better insights and generate hypotheses with respect to the potential benefits of statins. The results of ProfileChaser are shown in Table 4. The tested expression profiles were compared to the “statin users vs. non users” profile. The most significantly correlated profile was from Huntington’s disease patients, and the direction of effect was that “normal vs. presymptomatic/ symptomatic Huntington’s” correlated with statin users vs. non-users. This was followed by the profile of acute myeloid leukemia; control vs. acute myeloid leukemia profile correlated with statin vs. non-statin users profile. The third correlated profile was that of Parkinson’s disease, where the disease vs. control profile correlated with statin vs. non-statin users profile.

**Table 4:** ProfileChaser results of similar expression profiles.

GEO Dataset	Title	Subset 1 vs. Subset 2	Score	FDR
GDS1331	Huntington's disease: peripheral blood expression profile	normal vs symptomatic	0.54	0.010
GDS1059	Acute myeloid leukemia response to chemotherapy	normal vs AML	0.49	0.015
GDS2519	Early-stage Parkinson’s disease: whole blood	Parkinson's disease vs neurodegenerative disease control	0.46	0.018
GDS3073	Neutrophil response to aerobic exercise	before exercise vs after exercise	0.44	0.022
GDS3318	Sickle cell disease: platelets	sickle cell disease vs control	0.40	0.032
GDS1615	Ulcerative colitis and Crohn's disease comparison: peripheral blood mononuclear cells	Crohn's disease vs normal	0.39	0.035
GDS2362	Presymptomatic and symptomatic malaria: peripheral blood mononuclear cells	presymptomatic vs experimentally acquired	0.38	0.037
GDS3646	Celiac disease: primary leukocytes	healthy control vs celiac disease	0.36	0.044

GEO dataset: refers to the dataset accession number in GEO. Title: title of the gene expression study with correlated profile. Subset 1 vs. subset 2: the direction of effect in the correlated experiment as it relates to the statin signature in the current study for which the direction is statins users vs. non-users. Score: Weighted Pearson’s correlation between the statin DE genes and the identified profiles. FDR: false discovery rate

## Discussion

In the current study, a gene signature which correlated with statin use in the blood of COPD patients was identified. 25 genes were differentially expressed at 10% FDR. These genes were significantly enriched in pathways and biological processes related to cholesterol homeostasis and metabolism, consistent with statins’ mechanisms of action.

In hepatocytes, statins inhibit the 3-hydroxymethyl glutaryl coenzyme A (HMG-CoA) reductase enzyme; the rate-limiting step in cholesterol synthesis that converts HMG-CoA to mevalonic acid, a cholesterol precursor[40]. When cells are deprived of cholesterol, proteolytic cleavage generates the transcription factor SREBP-2 that translocates to the nucleus, binds and activates the promoters of SREBP-2-regulated genes involved in cholesterol biosynthesis such as HMG-CoA-synthase, HMG-CoA reductase, and cholesterol uptake genes including the low-density lipoprotein receptor (*LDLR*) [41]. In turn, the increased *LDLR* expression leads to more cellular uptake of LDL cholesterol, depleting it from the circulation. The other important transcription factor involved in cholesterol homeostasis is liver X receptor (LXR), which responds to elevated levels of cholesterol by up-regulating cholesterol efflux genes such as the ATP-binding cassette, sub-family A, member 1 (*ABCA1*), and ATP-binding cassette sub-family G member 1 (*ABCG1*) [42–44].

The statins’ blood gene signature identified in this study is reflective of this mechanism. Indeed, the most significantly associated gene was the *LDLR*, which was up-regulated among patients using statin. Of the strongly correlated genes, *ABCG1* was down-regulated with statin use, in line with previous reports [45]. Furthermore, sterol-C4-methyl oxidase-like (SC4MOL) and 24-dehydrocholesterol reductase (*DHCR24*, also known as seladin 1) were significantly up-regulated among statin users. *DHCR24* up-regulation has been previously reported with simvastatin treatment [46] and mediates the anti-inflammatory effects of reconstituted HDL [47]. Myosin regulatory light chain interacting protein (*MYLIP*) gene, also known as the inducible degrader of the LDLR (*IDOL*) was significantly down-regulated with statin use. Loss of function mutation in *MYLIP* has been identified in individuals with low LDL [48]. Furthermore, the ENCODE data which indicated SREBP-2 as the only transcription factor enriched among statins differentially expressed genes suggest that this pathway is orchestrated through this transcription factor.

The most significantly enriched pathways in the statin gene signature were cholesterol homeostasis and lipoprotein levels. Significant associations between serum lipid levels and lung function have been reported, albeit not always in the same direction [13–17]. Taken together, the differential gene expression results at the level of individual genes, pathways, and transcription factor enrichment strongly support the biological plausibility of the identified gene signature.

Comparing the statin-use signature with GEO profiles is an unbiased approach to identify shared expression profiles for different diseases, drugs, or perturbations. Concordant expression profiles leads to novel insights into the molecular mechanisms underlying a disease/drug of interest and generates hypotheses. Using ProfileChaser to find correlated profiles, a number of diseases were identified as having an expression profile that is similar to the statin use signature. The strongest correlation was with a Huntington’s disease (HD) expression profile[49]. The direction of effect was that HD controls vs. symptomatic or presymptomatic patient’s profiles correlate with statin user vs. non-users, suggesting a potential beneficial role for statins in HD patients. One study in rats have shown that statins, together with rofecoxib, exert a synergistic neuroprotective effect against malonic acid induced Huntington's disease and related cognitive dysfunction [50].

The second correlated expression profile with statin use was a signature of improved prognosis among pediatric acute myeloid leukemia patients (AML). In tumor cell lines, statins were shown to inhibit the growth of acute myeloid leukemia cells [51], to synergistically increase the killing of the human erythroleukemia cell line K562 by the chemotherapeutic agent cytosine arabinoside [52], and to inhibit the proliferation and cytotoxicity of a human leukemic natural killer cell line [53]. The third correlated signature with statin use was a signature of Parkinson’s disease (PD) patients when compared with healthy or neurodegenerative disease controls[54]. This suggests that statin may have an unfavorable effect on PD. A number of epidemiological studies have investigated the risk of PD in statin users with conflicting results (reviewed in [55,56]).

While there were no direct expression profile similarities or pathway enrichment in known respiratory disease or related phenotypes, the most significantly differentially expressed genes among statin users have been shown to play a role in lung pathophysiology (reviewed in [20]). In animal models, *Ldlr*-/- male mice (but not females) showed impaired alveologenesis [57]. In contrast, other studies have shown that *Ldlr*-/- mice fed with an atherogenic diet did not exhibit lung destruction or inflammatory changes, while airspace enlargement developed in the lungs of apolipoprotein E-deficient (*Apoe*(-/-)) mice exposed to the same diet[58]. *Abcga1*-/- mice have a pulmonary phenotype characterized by excess cholesterol in alveolar macrophages and type II cells [59–61]. *Abcga1*-/- mice also demonstrated an exaggerated inflammatory response to LPS and *K*. *pneumoniae* [62]. *CXCR2* is a chemokine receptor that is expressed by leukocytes, endothelial and epithelial cells, with IL-8 being the major ligand in humans[63]. *CXCR2* plays an important role in neutrophil homestasis [64,65], and increased expression of *CXCR2* has been reported in bronchial biopsies from COPD patients with severe exacerbations[66].

The current study has a number of limitations. First, information on the type and dose of statin was not captured in the ECLIPSE cohort. Studies have shown that different statins produce divergent gene expression profiles [67,68]. Second, gene expression is tissue specific, and hence it is possible that a similar approach in respiratory-relevant tissues such as lung tissue, or airway epithelial cells could lead to different results. Furthermore, *in vitro* and *in vivo* studies are needed to delineate the effect of statins in these tissues. Third, the results of this study are context specific, and as such different COPD cohorts may produce different results. For example, in COPD patients with evidence of existing systemic inflammation (such as elevated C reactive protein), statin use may produce a different expression profile. The same applies to the studies that showed correlated expression profiles with statins signature. Results from these studies need to be interpreted in the context of their respective cohort. In Parkinson’s disease, for instance, the low LDL cholesterol correlation with the disease may be a consequence rather than a cause of the disease. Future studies are warranted to investigate statins’ effects on these diseases. Finally, serum lipid levels were not available on ECLIPSE subjects, and it was not possible to correlate serum lipids with lung function and explore the impact of statin use on lipid levels and gene expression.

In conclusion, the current study has shown that statins have a significant effect on blood gene expression from COPD patients. Statins altered cholesterol homeostasis and metabolism pathways with no direct effect on known pathways related to respiratory diseases. The results suggest that reported benefits of statin use in COPD patients may mainly be driven by their effect on cholesterol homeostasis and the resulting cardiovascular protection. Further studies on the relevance of cholesterol homeostasis on lung pathophysiology in respiratory tissues such as lung or airway epithelium are warranted. The study highlights the use of gene expression profiling as a tool to inform prospective epidemiological studies and clinical trials.
